# Euler‐Top Gaussian Modes: Structured Beams From Quadratic Angular Momentum Dynamics

**DOI:** 10.1002/nap2.70077

**Published:** 2026-05-05

**Authors:** Mark R. Dennis, Kerr Maxwell

**Affiliations:** ^1^ School of Physics and Astronomy University of Birmingham Birmingham UK; ^2^ Centre for Doctoral Training in Topological Design University of Birmingham Birmingham UK; ^3^ H H Wills Physics Laboratory University of Bristol Bristol UK

## Abstract

We propose a new family of paraxial Gaussian modes, the Euler‐top Gaussian modes. Motivated by SU(2) angular momentum spin algebra, these modes are defined as eigenfunctions of a natural quadratic combination of spin‐like Fradkin–Stokes parameters $K=L^{2}-M_{+}^{2}$. Together with Hermite–Gaussian, Laguerre–Gaussian and Ince–Gaussian modes, these new modes complete the natural (quadratic) families of Gaussian eigenmodes, up to SU(2) transformations. Euler‐top Gaussian modes with positive eigenvalues have a real Laguerre–Gaussian appearance, and those with negative eigenvalues have a coupled Hermite–Gaussian appearance with fourfold symmetry. Certain other modes quantise a separatrix state between the two regimes. Their structure is motivated by paths on the Gaussian ray‐orbital Poincaré sphere, analogous to the polhode curves describing the angular momentum of an Euler top, that is, a classical rigid body with three different axes of inertia. Various properties of these new modes are considered, including their analogy to Lipkin–Meshkov–Glick modes from nuclear physics.

## Graphical abstract

A new family of structured Gaussian light beams are introduced: Euler‐top Gaussian modes. Their ray‐orbital paths on the Gaussian Poincaré sphere correspond to the polhodes of the Euler top in classical angular momentum theory. This geometric and algebraic construction reveals a nonseparable mode family extending the familiar Hermite‐, Laguerre‐ and Ince–Gaussian beams.

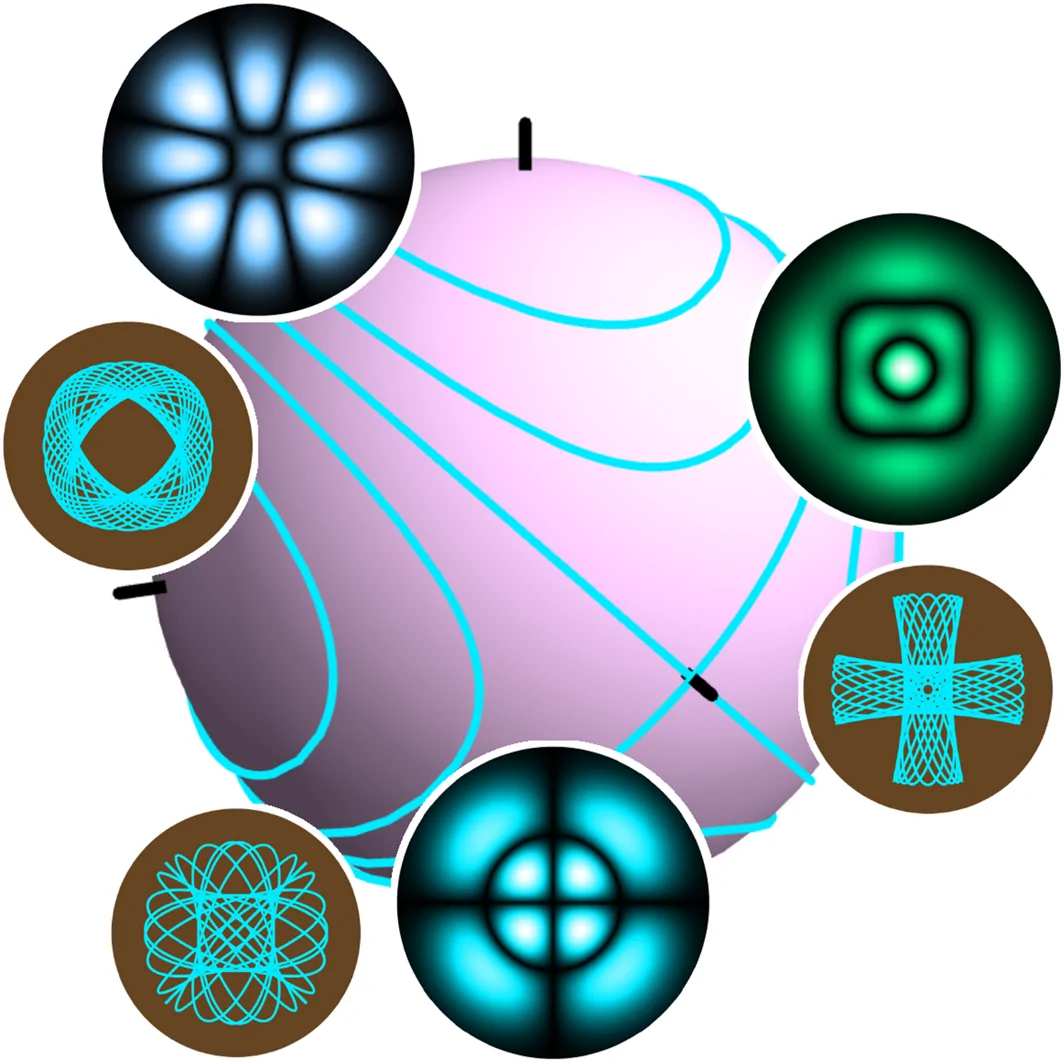

## 1 Introduction

Structured light has developed over the past few decades at the intersection of optical engineering, wave mechanics and topological optics [1, 2]. Families of paraxial scalar light beam wavefunctions are designed to exhibit topological and geometric structures, such as nodal lines and vortices; they are also eigenfunctions of well‐defined optical operators such as angular momentum [1, 2, 3, 4, 5, 6, 7]. The most familiar examples are *structured Gaussian modes* where solutions of the paraxial wave equation with Gaussian envelope are known to separate in exactly three transverse 2D orthogonal coordinate systems [8, 9]: Hermite–Gaussian (HG) modes in Cartesian coordinates x,y [4, 10], Laguerre–Gaussian (LG) modes in polar coordinates r,ϕ [3, 4] and Ince Gaussian (IG) modes in elliptic coordinates [11, 12].

Here we introduce a new mode family of structured Gaussian modes, motivated not by separation of variables, but by angular momentum algebra. We refer to these as ‘Euler‐top Gaussian’ (EG) modes because their definition is closely related to the classical dynamics of the Euler top: a rotating classical body whose three principal moments of inertia are all distinct [13, 14]. Because all paraxial structured Gaussian modes of fixed mode order share the same beam quality and Gouy phase, linear optical transformations map between them; algebraically, they form a finite‐dimensional family closed under quadratic optical observables/operators. Although not separable in a transverse coordinate system, the EG modes are exact eigenfunctions of a natural family of optical operators, and, up to a straightforward generalisation, complete the quadratic algebraic classification of Gaussian modes of fixed beam quality.

The explicit definition of the EG modes relies on the formal analogy between Gaussian modes, the 2D quantum harmonic oscillator and quantum spin states. These are all eigenfunctions of sets of angular momentum‐like operators (satisfying su(2) angular momentum commutation relations), underpinned by various Poincaré sphere‐like structures. For Gaussian modes, the sphere here does not represent polarisation, but rather the reduced phase space of the Hamiltonian ray dynamics, which, following the literature [12, 15, 16, 17, 18], we call the *ray‐orbital sphere*. We now summarise this picture, and define the EG modes in relation to the LG, HG and IG mode families.

Each structured Gaussian mode has a mode order N=0,1,2,… reflecting the fact that it is an eigenfunction of the *beam quality.*

(1) $$M^{2}=x^{2}+p_{x}^{2}+y^{2}+p_{y}^{2}=2H_{x}+2H_{y},$$

where $H_{x}=\frac{1}{2}\left(x^{2}+p_{x}^{2}\right)$ and $H_{y}=\frac{1}{2}\left(y^{2}+p_{y}^{2}\right)$. Here, x,y are transverse position coordinates and $p_{x},p_{y}$ are the corresponding momenta (i.e., transverse direction). The eigenvalue corresponding to each mode is proportional to N+1, that is, its well‐defined Gouy phase. For each N, the modes are N+1‐fold degenerate; the LG, HG and IG (and EG) basis sets are mutually related by inter‐basis expansions [4, 11, 12, 15].

The beam quality $M^{2}$ is twice the 2D harmonic oscillator Hamiltonian $H_{\text{HO}}=H_{x}+H_{y}$; as is well known, the structured Gaussian modes are equivalent to 2D quantum harmonic oscillator energy eigenstates [4, 6]. We note that there is some ambiguity in the notation: quadratic combinations of $x,y,p_{x},p_{y}$ can represent observables in 4D phase space, or operators acting on transverse beam amplitude wave packets. We will attempt to keep the meaning clear from context.

The N+1‐fold degenerate eigenspaces of the 2D quantum harmonic oscillator, correspond to the spin‐j irreducible representations of SU(2) with N=2j. This is the ‘Schwinger oscillator model’ [19], and we evidently have ${\left(\frac{1}{2}M^{2}\right)}^{2}=H_{\text{HO}}^{2}=4J^{2}$, where $J^{2}$ is the corresponding ‘total spin’ Casimir operator [1, 15, 16, 17].

This connection underpins the ‘Gaussian mode sphere’ [1, 4, 20, 21, 22, 23, 24]. LG HG, and generalised Laguerre–Hermite Gaussian (GG) modes (with mode order N=2j) are related by SU(2) rotations, with each N+1‐dimensional mode set represented by a point on the unit sphere. The N=1 $\left(j=\frac{1}{2}\right)$ ‘Poincaré sphere equivalent’ relates first‐order Gaussian modes, the Bloch sphere, and the standard Poincaré sphere (parametrising the classical 2D harmonic oscillator) [4, 15, 16, 17, 18, 20].

The classical ray‐orbital sphere itself underpins the Gaussian mode sphere. Each point on this ray‐orbital sphere is an *elliptic orbit* of rays crossing the focal plane with position x,y and direction $p_{x},p_{y}$ [15, 16, 17]. Its 1,2,3 directions correspond to angular momentum‐like *Stokes–Fradkin parameters* [12, 15, 16, 17, 18].

(2) $$\begin{array}{ll} M_{+} & =H_{x}-H_{y},\\ M_{\times } & =p_{x}p_{y}+xy,\\ L & =xp_{y}-yp_{x}. \end{array}$$

These are quadratic functions of optical phase space, analogous to half the Stokes parameters for the standard Poincaré sphere. As phase space observables (not operators), they satisfy the relation $H_{\text{HO}}^{2}=M_{+}^{2}+M_{\times }^{2}+L^{2}$, and they form a su(2) Poisson algebra, with Poisson brackets $\left\{M_{+},M_{\times }\right\}=2L$, $\left\{M_{\times },L\right\}=2M_{+}$, $\left\{L,M_{+}\right\}=2M_{\times }$ [15, 16, 17].

When $\left(M_{+},M_{\times },L\right)$ are considered as operators, each Gaussian mode can be considered as built up from these ellipses, on parametrised *Poincaré paths* on the ray‐orbital sphere [15, 16, 17]. This process of constructing Gaussian modes from ellipses of rays can be considered as a ‘quantisation’ [25] of this Poincaré path of ray‐orbital ellipses.

To avoid confusion, we will distinguish the dynamical, physical Stokes–Fradkin triplet $\left(M_{+},M_{\times },L\right)$ from the triplet of algebraic angular momentum‐like operators $J=\left(J_{1},J_{2},J_{3}\right)$, although they have similar roles (and algebraically differ by a factor of 2). The latter triplet satisfies the usual angular momentum commutation relations: $\left[J_{j},J_{k}\right]=i{\;\varepsilon }_{ijk}\;J_{i}$, for i,j,k=1,2,3. We refer to $J_{1},J_{2},J_{3}$ as ‘pseudo‐AM’ operators rather than AM (angular momentum) operators: they encode the spin‐like algebra of the quadratic Gaussian structure, not physical angular momentum in real space (except for L and $J_{3}$).

In this notation, LG modes ${LG}_{\ell ,p}\left(r,\phi \right)$ are eigenfunctions of the AM operator $2J_{3}$ with eigenvalues ℓ, the azimuthal quantum number $p=\frac{1}{2}\left(N-|\ell |\right)$ is the radial quantum number, corresponding to L. The HG modes ${HG}_{m,n}\left(x,y\right)$ with quantum numbers m,n in x,y, are eigenfunctions of $2J_{1}$, corresponding to $M_{+}$, with eigenvalues m−n, and their 45° rotation to $2J_{2}$ and $M_{\times }$. Their eigenvalues are μ=−N,−N+2,…,N, with Poincaré paths on the ray‐orbital sphere that are circles centred on the 3, 1 and 2 axes respectively, enclosing proportional areas.

The IG modes arise slightly differently, determined by elliptic separability. The underlying dynamical quantity is the nonlinear combination $I=L^{2}+f^{2}M_{+}$, where f≥0 is the focal parameter of the elliptic coordinate system. The IG modes are therefore eigenfunctions of the corresponding *Ince operator* [9, 12, 26],

(3) $$\hat{I}=4J_{3}^{2}+2f^{2}J_{1},$$

as well as eigenfunctions of $H_{\text{HO}}=\frac{1}{2}M^{2}$ with eigenvalues N+1. As f→0, they become (real) LG‐like, and as f→∞, HG‐like. On the ray‐orbital sphere, Poincaré paths are the contours of the observable I. Geometrically, these correspond to the intersections of the sphere with an off‐centre cylinder [12, 26]. Due to the nonlinearity of I, the eigenvalues $\iota_{\mu }$ of I are nontrivial, but still labelled by μ, μ=−N,−N+2,…,N [12].

We are now in a position to define the Euler‐top Gaussian modes. They correspond to the observable andare eigenfunctions of the operator.

(4) $$K=L^{2}-M_{+}^{2},\;\hat{K}=4\left(J_{3}^{2}-J_{1}^{2}\right).$$

In 4D phase space, K involves a quadratic combination of $x,y,p_{x},p_{y}$, and is not separable. In the spin analogy, $\hat{K}$ is quadratic in the $J_{i}$ operators, so possesses similar simple but nontrivial algebraic structure as the Ince operator: algebraically tractable in terms of ladder operators, but more subtle than eigenfunctions of linear combinations of $\left(J_{1},J_{2},J_{3}\right)$ such as LG, HG and GG modes.

The EG modes, as simultaneous eigenfunctions of the commuting operators $H_{\text{HO}}$ and $\hat{K}$, have many attractive properties, and have well‐defined mode order N and Gouy phase. The operator $\hat{K}$, similar to the Ince operator $\hat{I}$, is a nonlinear quadratic combination of components of J, with eigenfunctions and eigenvalues which are algebraically tractable but has a more subtle structure than those of the spin‐like linear combinations of J underlying LG, HG and GG modes. In the ray‐orbital Poincaré sphere picture, their Poincaré paths are contours of $K=L^{2}-M_{+}^{2}$: pairs of closed curves analogous to the precessing motion of the angular velocity vector of the Euler top [13, 14], which are called *polhodes* in Poinsot's classical theory [27].

The eigenvalues of $\hat{K}$, which we denote $\kappa_{N,\mu }$, have three regimes:

- $\kappa_{N,\mu }>0$: modes are dominated by squared angular momentum $L^{2}$, and are LG‐like;
- $\kappa_{N,\mu }<0$: modes are dominated by $M_{+}^{2}$, and are HG‐like;
- $\kappa_{N,\mu }=0$: a topological transition where the two regimes meet at a separatrix, corresponding to the unstable rotation of a rigid body about its intermediate axis. When N is even, there is a well‐defined separatrix EG mode at $\kappa_{N,0}=0$.

Intensity patterns for the lowest EG modes are shown in Figure 1.

**FIGURE 1:**
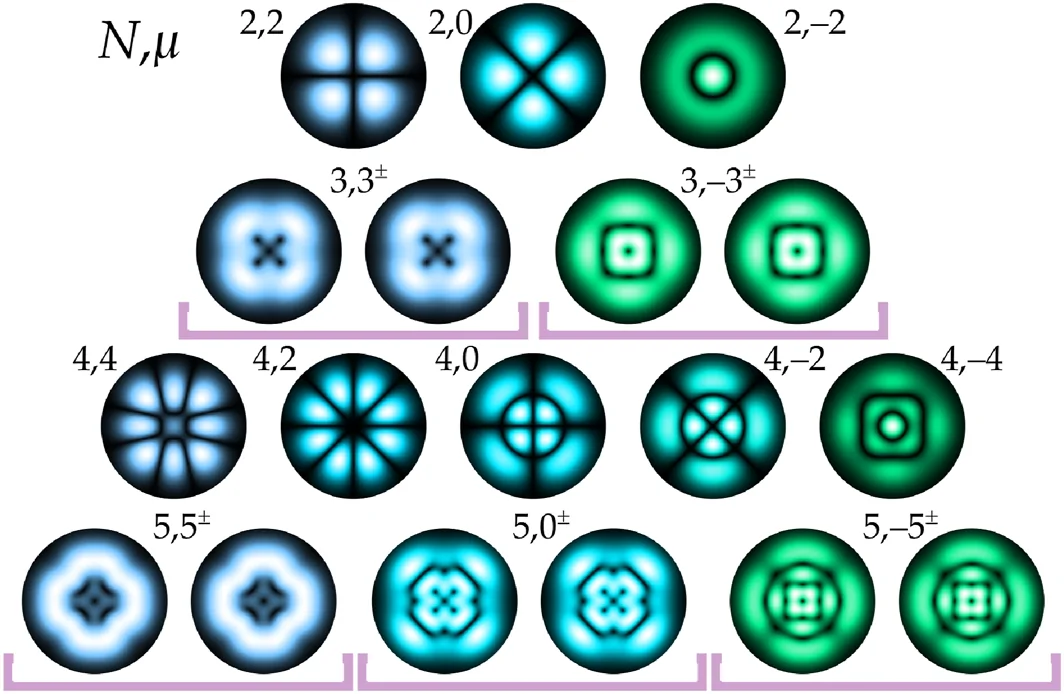
Euler‐top Gaussian modes. Rows are labelled by N=2,3,4,5. When N is even, modes with μ=N,N−2,…,−N, with LG‐like (HG‐like) modes represented in blue (green), have positive μ (negative μ). In each case, the separatrix state μ=0 is plotted in the middle in cyan. For odd N, the states are doubly (Kramers) degenerate and whose amplitudes are mutually conjugate (labelled ±), as discussed in Section 3.2.

In the next section, we develop an understanding of EG modes through polhode Poincaré paths on the ray‐orbital sphere, by analogy with those of the LG, HG, and IG modes, and through their connection with the Euler top. We then consider the spin algebra representation of $\hat{K}$ to calculate the EG modes directly, providing insight into their geometric and physical properties. We conclude with a brief discussion of their wider significance in optics and as a model wave‐mechanical system.

## 2 Gaussian Ray‐Orbital Sphere Geometry

We now motivate the Euler‐top Gaussian (EG) modes by comparing them with the separable HG, LG and IG families on the ray‐orbital Poincaré sphere. Each mode family is associated with a phase space observable, whose contours provide the modes' Poincaré path. Every mode is determined by one or more curves on ray‐orbital sphere, tracing out a family of elliptic ray orbits whose quantisation gives the mode [15, 16, 17, 18]. Although we focus here on the LG and HG modes, determined by L and $M_{+}$ (and $J_{3}$ and $J_{1}$), respectively, any linear superposition of $L,M_{+},M_{\times }$ determines a set of GG modes [1, 4, 18, 20, 21, 22, 23, 24], which we emphasise less in this work as these are accessed by SU(2) rotations from the LG and HG mode sets. Readers uninterested in the elliptic ray orbit picture may largely omit this section.

From quantisation of the rays [16, 17, 18], the ray‐orbital sphere naturally has radius $\sqrt{N+1}$, and each point on this sphere corresponds to an ‘elliptic orbit’ Gaussian coherent state ${ϒ}_{N,e}\left(r\right)$ analogous to a spin coherent state. Each of these states is parametrised by a Jones‐like complex vector e=q+ip with $|e{|}^{2}=N+1$. ${ϒ}_{N,e}\left(r\right)$ itself is a smooth (integral) superposition of fundamental Gaussian beams with transverse centre (position) Re{eexp(−iτ)} and tilt (momentum) Im{eexp(−iτ)}, as τ varies from 0 to 2π, and weighted by amplitudes exp(i(N+1)τ). It does not play a fundamental role in our construction, but is included here for completeness.

A structured Gaussian mode of order N is constructed from a closed Poincaré path, which is a contour of an arbitrary function $F\left(M_{+},M_{\times },L\right)$ on the ray‐orbital sphere. Along this path, the coherent states ${ϒ}_{N,e}\left(r\right)$ are smoothly superposed, weighted by a Pancharatnam–Berry phase proportional to the solid angle enclosed by the path [28]. The quantisation rule is given by the following equation:

(5) $$\Omega_{\mu ,N}=\frac{2\pi \mu }{N+2},$$

for μ=0,±2,…±N (even N) or μ=±1,±3,…±N (odd N), ensures that the accumulated geometric phase is an integer multiple of 2π, when $F\left(M_{+},M_{\times },L\right)$ is a linear combination. This picture provides a geometric quantisation of the integrable invariant torus in the four‐dimensional phase space of the 2D harmonic oscillator [29, 30].

When $\hat{F}=2\left(a_{1}J_{1}+a_{2}J_{2}+a_{3}J_{3}\right)=2a\cdot J$ for real unit 3‐vector a, the corresponding LG, HG and GG modes can be constructed directly from the spin coherent state Jones vector [17, 18] and quantisation is exact [31]. When $F\left(M_{+},M_{\times },L\right)$ is a higher‐order polynomial or a more general function, there is not such a clear connection in general between the modes and the contours of F as Poincaré paths.

Nevertheless, choices of F which are quadratic, such as I and K underlying the IG and EG mode families, respectively, have a simple enough contour structure to be identified with the exact eigenstructure of the corresponding $\hat{F}$. In all these cases, the coherent state superposition helps the understanding of the wave structure: each mode is associated with a one‐parameter family of ellipses on the ray‐orbital sphere encoding the symmetry of the corresponding observable F. This provides a unified interpretation for the LG, HG, GG, IG and the new EG family of structured Gaussian modes. Up to SU(2) rotation, combinations of I and K generate the whole quadratic algebra of invariants of Gaussian modes [32].

### 2.1 LG, HG and GG Modes

For LG modes (HG modes), the operator $\hat{F}$ is $2J_{3}$ $\left(2J_{1}\right)$. The Poincaré paths are contours of L $\left(M_{+}\right)$: coaxial circles with axis along the 3‐ (1‐)axis, with latitudes set at μ/(N+2) from $\Omega_{\mu ,N}$. For LG modes, μ=ℓ (i.e., eigenvalue of $2J_{3}$, which is the usual azimuthal quantum number); for HG modes, μ=m−n (i.e., eigenvalues of $2J_{1}$).

Circular contours in the 1,2‐ and 2,3‐ planes give the LG and HG Poincaré paths shown in Figure 2a. Their projections into the 1,2‐plane, under a natural squaring conformal transformation [16], are related to the caustics of the corresponding ellipse families and modes, shown in Figure 2b. HG paths are straight lines in the 1,2‐plane, and the ellipse family caustics are rectangular (reflecting Cartesian separability). LG paths are circles, and the families of ellipses are enclosed by concentric circular caustics, reflecting polar separability.

**FIGURE 2:**
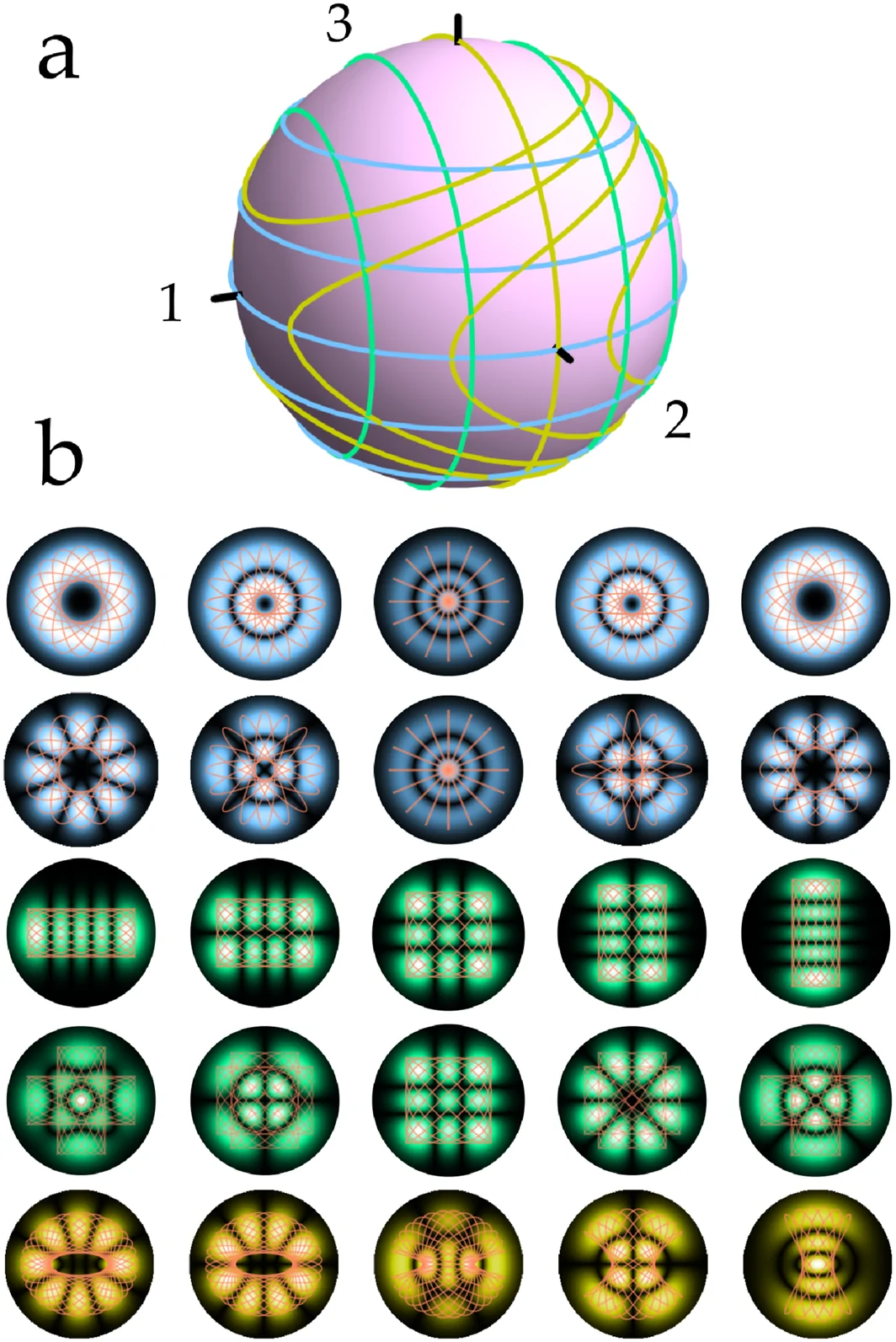
Gaussian mode families and the N=4 ray‐orbital Poincaré sphere. (a) Sphere with Poincaré paths: LG (blue), HG (green) and IG (with f=0.2, yellow). (b) N=4 mode intensities. Rows represent eigenfunctions for $J_{3},J_{3}^{2},J_{1},J_{1}^{2},\hat{I}$, respectively. Rows 1, 3, and 5 are standard LG, HG and IG modes, whereas rows 2 and 4 are even (left) and odd (right) squared LG and HG modes. Ellipses from the corresponding Poincaré paths are superposed in orange.

When F is alternatively defined via a linear combination $F=a\cdot \left(M_{+},M_{\times },L\right)$, for real vector a, the Poincaré paths are circles with axis a subtending the same $\Omega_{\mu ,N}$ areas as LG and HG modes [17, 18]. The GG modes are the eigenfunctions of the corresponding operator 2J⋅a, but outside the 1,2,3 axes, are not separable [1, 4, 18, 20, 21, 22, 23, 24]. They can nonetheless be expressed as linear superpositions of LG or HG modes weighted by appropriate matrix elements of the SU(2) rotation; their ellipse families represent some arbitrary projection of the phase space torus [15, 17].

### 2.2 Squared LG and HG Modes

As an eigenfunction of $2J_{3}$, each complex LG mode ${LG}_{\ell ,p}\left(r,\phi \right)$ contains a complex phase term exp(iℓϕ). Real LG modes [10], which we here call *squared LG modes*, are proportional to cosℓϕ and sinℓϕ, and are eigenfunctions of the degenerate, quadratic $J_{3}^{2}$. They have two circular Poincaré paths with equal and opposite $\ell^{2}=\mu^{2}$; $J_{3}^{2}$ therefore couples the LG modes with labels N,±μ. The associated ray ellipse orbits have identical shapes but opposite orientations (analogous to left‐and right‐handed elliptic polarisations); the resultant modes are real‐valued interference patterns, as shown in Figure 2b, with fourfold rotational symmetry. Furthermore, the eigenvalues $\ell^{2}$ are no longer directly related to the areas $\Omega_{\ell ,N}$.

Similarly, *squared HG modes* are eigenfunctions of the quadratic operator $J_{1}^{2}$. They are of even and odd types, proportional to $H_{m}\left(x\right)H_{n}\left(y\right)\pm H_{n}\left(x\right)H_{m}\left(y\right)$; squared HG modes couple the standard HG modes labelled by N,±μ. Again, they can be constructed from pairs of Poincaré paths, now along the 1‐axis. The squared HG modes are real‐valued, and have nodal patterns which are, approximately, circles and radial lines more commonly associated with real‐valued LG modes, as can be seen in Figure 2b.

### 2.3 IG Modes

As discussed above, the IG modes are separated in elliptic coordinates, and thus do not have the simple spin‐like SU(2) structure characterising the LG, HG and GG modes. The underlying Ince operator $\hat{I}$ in Equation (3) is a mixture of linear and quadratic pseudo‐AM operators. As $\hat{I}$ depends on the interfocal parameter f, the IG modes are in fact a 1‐parameter family interpolating between HG modes (f→∞) and real squared LG modes (f=0).

On the ray‐orbital sphere, the Poincaré paths are the contours of $F=I=L^{2}+fM_{+}$, which are intersections of the sphere with cylinders parallel to but offset from the 3‐axis. When f is small and eigenvalues ι of $\hat{I}$ are large, IG modes are squared LG‐like, corresponding to those intersections comprised of two loops; in contrast, in the HG‐like regime (not squared HG), there is a single loop intersection [12]. A notable feature of IG modes is a *topological transition* [26]: in between the two regimes, the contours of F have a self‐intersection, where the sphere is tangent to the cylinder.

The classical dynamics of I are those of the ‘Ince oscillator’: the phase portrait of a nonlinear pendulum wrapped around the sphere [12, 26]. The LG regime has two curves and is a ‘rotating’ pendulum orbit; the modes are perturbations of squared LG modes. The HG regime (one curve) is a ‘oscillating’ pendulum orbits, and the modes are perturbations of regular HG modes. The azimuth‐doubling inherent in the ray Poincaré construction implies that the IG Poincaré paths, displaced towards the +1‐direction, possess the characteristic π‐rotational symmetry of elliptic coordinates in the transverse plane.

However, due to nonlinearity, the eigenvalues of $\hat{I}$ do not correspond to the quantised areas subtended by contours of I [12]. An example of such contours for N=2 is shown in Figure 2a: as I decreases, pairs of LG‐like contours merge at a saddle point in the 1‐direction to a single HG‐like contour. The bottom row of Figure 2b shows the corresponding IG modes, with a reasonable qualitative match to the ellipse families from the contours.

### 2.4 EG Modes

The observable $F=K=L^{2}-M_{+}^{2}$ is a quadratic form. On the ray‐orbital sphere, the 1‐ and 3‐axes are weighted equally but with opposite sign, and the 2‐axis is neutral. When a multiple of the constant $H_{\text{HO}}^{2}=M_{+}^{2}+M_{\times }^{2}+L^{2}$ is added to K, the contours correspond to the intersections of the sphere with a harmonically balanced ellipsoid.

This corresponds exactly to the interpretation of the path of the angular velocity vector of an Euler top in its body frame. The resulting polhode curves are found from the intersection of the energy sphere with the inertia ellipsoid [13, 14, 27]. The 3‐axis (1‐axis) corresponds to the largest (smallest) moment of inertia, and the 2‐axis to the intermediate, unstable axis. Polhodes are not perfect circles, giving the classical ‘wobble’ around pure circular precession about the stable axes. These polhode curves on the sphere, and corresponding ellipse families, are shown in Figure 3.

**FIGURE 3:**
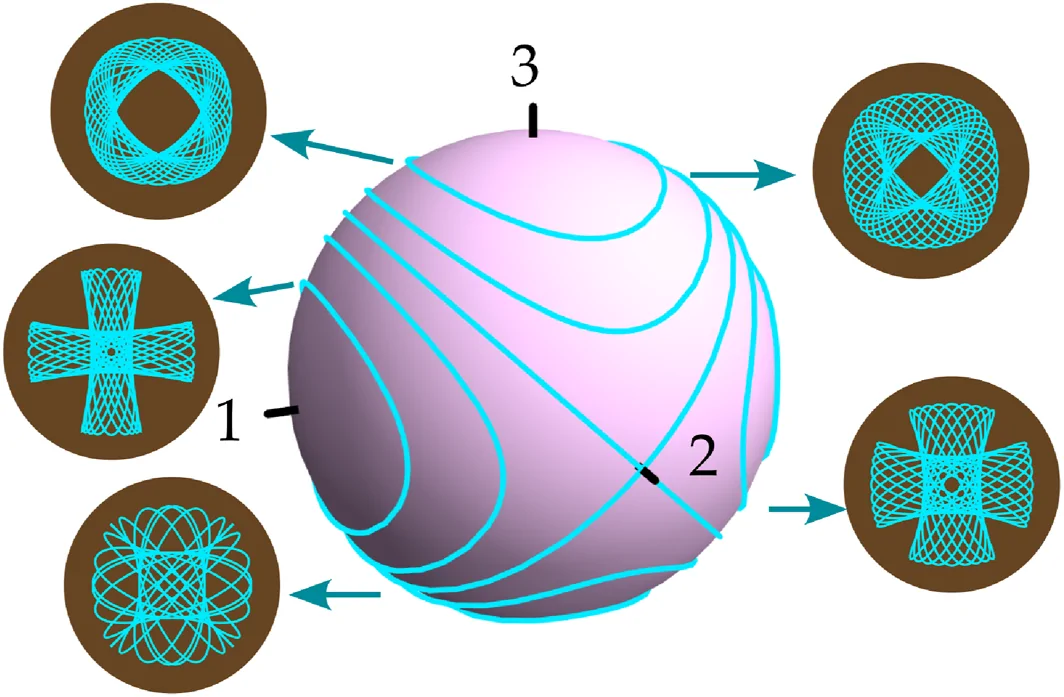
Polhode Poincaré paths. The curves on the ray‐orbital Poincaré sphere are polhodes corresponding to the eigenvalues $\kappa_{N,\mu }$ of K, which occur in opposite pairs except the self‐intersecting separatrix path. Each polhode pair corresponds to a family of ellipses along the Poincaré path: paths around the 3‐axis represent an LG‐like ‘squircle’ geometry (two upper insets), and those around the 1‐axis a HG‐like ‘Maltese cross’ (two middle insets). The ellipse family corresponding to the separatrix path sits between the characteristic squircle and maltese cross patterns (bottom inset).

Therefore K corresponds to a parameter‐free, balanced quadrupolar dynamical invariant encoding this anisotropic inertia structure. In 1,2,3‐space, the K contours have the dihedral symmetry of an ellipsoid, together with an inversion symmetry ±K when 1↔3 axes are exchanged.

When K>0, the loops lie in the upper and lower 3‐hemispheres around the 3‐axis. In this squared LG regime, these two polhodes on the ray‐orbital sphere correspond to ellipse families with nearly circular caustics. However, they are not squared LG functions: the polhode ‘wobble’ gives ellipse families with a fourfold symmetric ‘squircle’ structure [33, 34]. This suggests EG modes with positive eigenvalues κ>0 resemble squared LG modes, but deformed. The polhode symmetry endows them with a fourfold rotational symmetry, reflected in the plots in Figure 1.

For K<0, the polhode loops lie in the ±1‐directions. Each polhode loop gives an ellipse family bounded by a distorted rectangle (the distortion again due to the ‘wobble’). The net ‘Maltese cross’ structure has fourfold symmetry, again characteristic of azimuth doubling from the near‐elliptical polhodes around the 1 axis. This suggests EG modes with negative eigenvalue κ<0 are perturbations of squared HG modes, again reflected in the plots in Figure 1.

When K=0, the ellipsoid becomes tangent to the sphere at saddle points on the ±2‐axes, corresponding to meetings of a four‐arc *separatrix*: the net contour is a pair of geodesic circles intersecting on the ±2‐axis, and equidistant from the 1,3‐axes. In the classical intermediate axis theorem (also known as Poinsot instability, the ‘tennis racket theorem’ or the Dzhanibekov effect), rotation about the 2‐axis degenerates into a rotation about the 1‐ or 3‐axis. This reflects the instability of this separatrix contour, which for the EG modes is a topological transition between squared HG‐like and squared LG‐like regimes.

The balancing in K between $L^{2}$ and $M_{+}^{2}$ guarantees that the separatrix occurs symmetrically at K=0. The ellipse family whose Poincaré path is the separatrix contour, shown in the lower inset of Figure 3, is a transitional pattern interpolating between the squircle‐like and cross‐like caustics characteristic of the two polhode regimes, again with fourfold symmetry. This transitional state is realised by EG modes with even N and $\kappa_{N,0}=0$, visible in the N=2,4 cases in Figure 1.

A detailed semiclassical analysis of the EG polhode caustics and coherent state superposition, analogous to that of IG rays [12], requires elaborate elliptic integrals and is outside the scope of this paper. As K is quadratic, the exact eigenvalues of $\hat{K}$ do not correspond to quantised polhode areas.

However, such an analysis is unnecessary to calculate the EG modes themselves. The EG operator $\hat{K}$ may be analysed directly using quantum spin algebra, yielding both $\kappa_{N,\mu }$ eigenvalues and coefficients for their expression as LG or HG superpositions (like the nonseparable GG mode). This will be considered in the next section.

On the ray‐orbital sphere, the Poincaré paths of the LG, HG, IG and EG modes are all the intersections of the sphere with quadric surfaces, aligned with the 1,2,3‐axes. Projections of these curves into the 2,3‐ and 1,2‐ planes are shown in Figure 4. In the 1,2‐plane, the Ince paths are off‐centre circles, mapping to ellipses and hyperbolas [12], but in the 1,3‐plane they are parabolas, reminiscent of the ray structure of Airy beams. The polhode paths for the EG modes resemble the confocal ellipses of elliptic coordinates, but under the squaring transformation, these have fourfold rotational symmetry, similar to the ellipse families in Figure 3.

**FIGURE 4:**
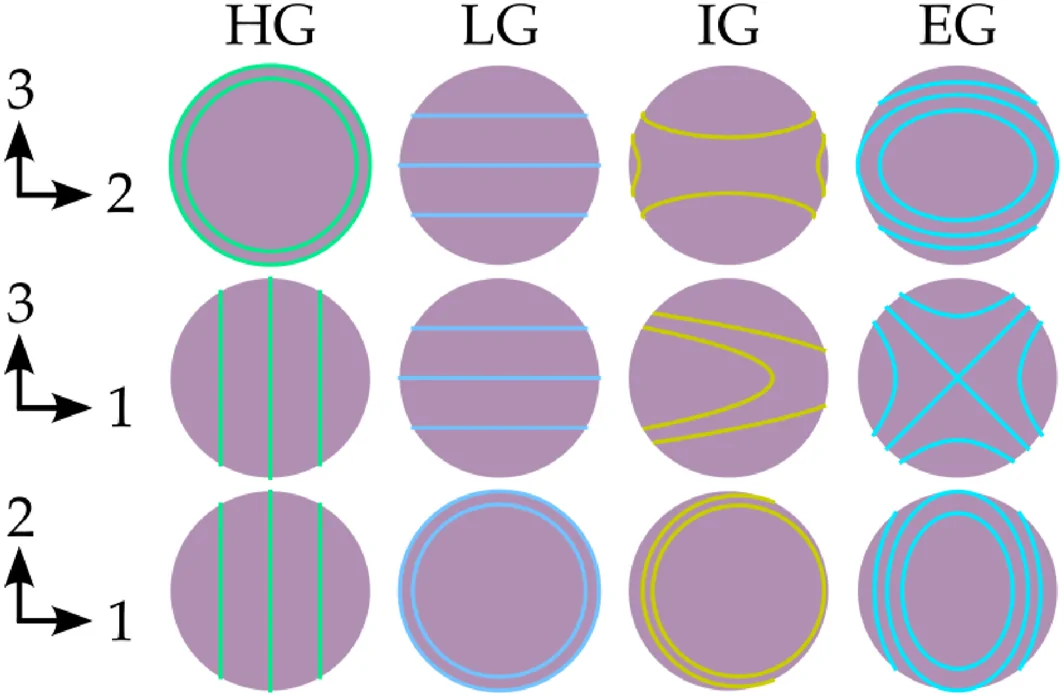
Projected paths on the ray‐orbital sphere. Rows correspond to projecting in the 1, 2 and 3‐directions, with columns corresponding to the N=3 Poincaré paths for LG, HG, IG (f=1/10) and EG modes.

## 3 Construction and Description of EG Modes

In this section, we construct EG modes as superpositions of LG modes. As with every Gaussian mode family, they are a set of eigenstates of an appropriate operator $\hat{F}=F\left(2J_{1},2J_{2},2J_{3}\right)$ corresponding to a function of Fradkin–Stokes parameters $F\left(M_{+},M_{\times },L\right)$. Thus in the quantum spin language, the EG modes are eigenstates of the SU(2) Casimir operator $J^{2}$, whose eigenfunctions are N+1‐fold degenerate for N=2j=0,1,2,…. The operator $\hat{K}$ in Equation (4) commutes with $J^{2}$, and its eigenfunctions provide a basis for each of the N+1‐dimensional Gaussian mode spaces.

We choose LG modes as quantum algebra is more familiar in terms of eigenfunctions of the 3‐direction, though the same construction using a basis of HG modes (or, indeed, GG modes) is equivalent. Our derivation, though complete, will not describe all the algebraic steps in detail; these can easily be filled in using standard quantum spin algebra, whose background and techniques are described in standard textbooks [35, 36]. In the quantum spin algebra, the spin component labels in the 3‐direction are m=−j,−j+1,…,j. In optics language, the role of m is taken by the angular momentum number ℓ of the LG modes, with $m=\frac{1}{2}\ell$.

The symmetries of K ensure the eigenmodes of $\hat{K}$ inherit features of the polhode contours. The 1↔3,±K symmetry ensures that positive and negative eigenvalues are paired, $\kappa_{N,\mu }=-\kappa_{N,-\mu }$, with even N having a zero eigenvalue $\kappa_{N,0}=0$ corresponding to the separatrix orbit.

### 3.1 EG Mode Algebra

We now construct the EG modes explicitly. As with all structured Gaussian beam eigenfunctions of operators built from pseudo‐AM operators, the EG modes lie in irreducible representations of SU(2) [4, 21, 24, 35]. For a given mode order N, the Casimir operator is given by:

(6) $$J^{2}=J\cdot J=J_{1}^{2}+J_{2}^{2}+J_{3}^{2}=\frac{1}{4}H_{\text{HO}}^{2}=\frac{1}{4}{\left(\frac{1}{2}M^{2}\right)}^{2},$$

where eigenvalues j(j+1) for j=N/2, and N+1‐dimensional degenerate subspaces.

Since the Euler top operator $\hat{K}$ commutes with $J^{2}$ but not $J_{3}$, its eigenfunctions define a new basis for this (2j+1)‐dimensional Gaussian mode subspace. For fixed N, $\hat{K}$ becomes ${\hat{K}}_{N}$, an (N+1)×(N+1) matrix, and we find the EG modes as superpositions of LG modes weighted by the components of the eigenvectors of ${\hat{K}}_{N}$.

We write the simultaneous eigenstate of $J^{2}$ and $J_{3}$ in quantum spin language as |j,m〉, with m=−j,−j+1,…,j. These correspond to LG modes with ℓ=2m and radial index p=j−|m|. Phase conventions are unimportant to these calculations due to the quadratic nature of $\hat{K}$.

In the |j,m〉 basis,

(7) $$J_{3}=diag\left\{j,\left(j-1\right),\text{…},-j\right\},$$

(8) $$J_{3}^{2}=diag\left\{j^{2},{\left(j-1\right)}^{2},\text{…},j^{2}\right\},$$

so $J_{3}^{2}$ does not distinguish between |j,±m〉.

In terms of raising and lowering operators, $J_{1}=\frac{1}{2}\left(J_{+}+J_{-}\right)$, therefore,

(9) $$J_{3}^{2}=\frac{1}{4}\left(J_{+}^{2}+J_{-}^{2}+J_{+}J_{-}+J_{-}J_{+}\right).$$

The mixed terms

(10) $$J_{\pm }J_{\mp }=J^{2}-J_{3}^{2}\pm J_{3}$$

are diagonal, and the double‐raising and double‐lowering terms couple m with m±2 terms, with easy‐to‐calculate matrix elements.

Substituting these into ${\hat{K}}_{N}$ gives the following compact expression:

(11) $${\hat{K}}_{N}=6J_{3}^{2}-2J^{2}-\left(J_{+}^{2}+J_{-}^{2}\right),$$

with nonzero matrix elements

(12) $$\begin{array}{ll} \langle j,m|{\hat{K}}_{N}|j,m\rangle & =6m^{2}-2j\left(j+1\right),\\ \langle j,m\pm 2|{\hat{K}}_{N}|j,m\rangle & =\sqrt{\left(j\mp m\right)\left(j+1\pm m\right)}\sqrt{\left(j+2\pm m\right)\left(j-2\mp m\right)}, \end{array}$$

where as usual j=N/2 and m=ℓ/2.

As m is coupled only to m or m±2, the off‐diagonal elements couple the ℓ,ℓ±4 LG states; this is the algebraic justification of the fourfold EG symmetry anticipated in Section 2.4. When N is even, the ${\hat{K}}_{N}$ matrix splits naturally into odd and even m blocks (the latter including the zero eigenvalue), and into two equivalent but distinct ±N blocks when N is odd. All of these matrices are tridiagonal in steps of Δm=±2, therefore, eigenfunctions and eigenvectors may readily be found numerically for larger matrices.

### 3.2 Description of EG Modes

We are now in a position to discuss the EG modes shown in Figure 1 in detail, based on the calculations of the previous section. The EG modes are superpositions of LG modes with coefficients given by the eigenvectors of the ${\hat{K}}_{N}$ matrix described in Equation (12). The LG modes are, explicitly [3, 4], which are given by where, for completeness, we write as

(13) $${LG}_{\ell ,p}\left(r,\phi \right)=\sqrt{\frac{p!}{\pi \left(|\ell |+p\right)!}}r^{\left|\ell \right|}\;\exp\left(i\ell \phi -\frac{1}{2}r^{2}\right)L_{p}^{\left|\ell \right|}\left(r^{2}\right).$$

Because $\hat{K}$ is a quadratic combination of $J_{3}$ and $J_{1}$, it cannot distinguish the two states of N=1, and indeed ${\hat{K}}_{N=1}=0$.

The first nontrivial case is N=2, where the 3×3 representation of ${\hat{K}}_{N=2}$ is given by the following equation:

(14) $${\hat{K}}_{N=2}=\frac{1}{4}\left(\begin{array}{ccc} 1 & 0 & -1\\ 0 & -2 & 0\\ -1 & 0 & 1 \end{array}\right).$$

The ℓ=0 state decouples from the ℓ=±2 pair; this is the HG‐like ${EG}_{N=2,\mu =-2}$ state corresponding to ${LG}_{\ell =0,p=1}$ in Figure 1, with eigenvalue $\kappa_{2,\pm 2}=-\frac{1}{2}$. The other two N=2 EG modes are ${LG}_{2,0}\pm {LG}_{-2,0}$, yielding the LG‐like $\left(\kappa_{2,2}=\frac{1}{2}\right)$ and separatrix $\left(\kappa_{2,0}=0\right)$ modes respectively. Although this case is too small to show the rich geometry of the EG family, it already exhibits the essential symmetries for even N: LG‐like and HG‐like modes occur as $\kappa_{N,\pm \mu }=\pm \;\kappa_{N,\mu }$ pairs, and the separatrix state with $\kappa_{N,0}=0$ sits exactly between them.

For even N, the spectrum of ${\hat{K}}_{N}$ consists of a symmetric ladder of eigenvalues $0,\pm \;\kappa_{N,2},\text{…},\pm \;\kappa_{N,N}$. The matrix naturally breaks into two sectors: one involving ℓ=0,±4,±8,… and the other with ℓ=±2,±6,…, guaranteeing that EG modes with even N modes are real‐valued with fourfold symmetry. The central state with $\kappa_{N,0}=0$ is the separatrix mode whose Poincaré path passes through the ±2‐axes of the ray‐orbital sphere. Their intensity patterns contain nonseparable nodal lines:

- For large $\kappa_{N,\mu }>0$, the modes resemble real LG‐like patterns with nearly radial nodal lines, and whose circular caustics are replaced by the squircle‐like traces similar to the corresponding ellipse families loops expected from the ±3‐axis polhode Poincaré paths.
- As $\kappa_{N,\mu }$ decreases, pairs of radial lines are replaced by squircle‐like nodal loops. At κ=0, the number of squircular nodes equals twice the radial line count.
- For $\kappa_{N,\mu }<0$, the radial lines are replaced by more squircle‐like nodal loops, reducing their value of $L^{2}$; these near square patterns correspond to the Maltese‐cross ellipse families from the ±1‐axis polhodes.

For odd N, the N+1‐dimensional space splits into two independent but identical blocks because the Δℓ=±4 selection rule preserves the parity of ℓ/2=m. Each block contains (N+1)/2 states. As a result, in this case, every eigenvalue of $\hat{K}$ is twofold degenerate, corresponding to a Kramers‐like doubling arising from the ℓ=±N parity sectors of the Gaussian modes (rather than from time‐reversal symmetry of half‐integer spins in standard quantum mechanics [35, 36]). The higher, odd N HG‐like EG modes tend to have a checkerboard array of vortices close to the beam axis, as is visible in Figure 1.

Odd‐N EG modes necessarily carry nonzero orbital angular momentum. Near the beam axis, their intensity patterns form approximately square arrays of vortices reflecting the fourfold symmetry of the ellipse families from polhode Poincaré paths; further from the axis, the patterns become more intricate combinations of squircle‐like and cross‐like features. The orbital angular momentum and vortex circulations are reversed in the two Kramers‐like families of EG modes.

A distinctive feature of all the EG modes is that they inherit a fourfold azimuthal symmetry from the Δm=±2 coupling in the K matrix, geometrically related to the twofold dihedral symmetry of the polhode curves on the ray‐orbital sphere. This symmetry becomes especially transparent when the modes are plotted as functions of ϕ/2 rather than ϕ, as in Figure 5. The even‐N modes have nodal lines along the 1‐ and 2‐ axes, or along approximately elliptical or hyperbolic arcs; in addition, the odd‐N modes have rows of vortices of alternating sign (from the vortex arrays). This ‘half‐azimuth’ viewpoint gives a clear visual interpretation of how EG modes arise from quantising the classical polhodes curves, providing a natural hybrid of the real focal plane and ray‐orbital sphere representations in which their underlying symmetry becomes manifest.

**FIGURE 5:**
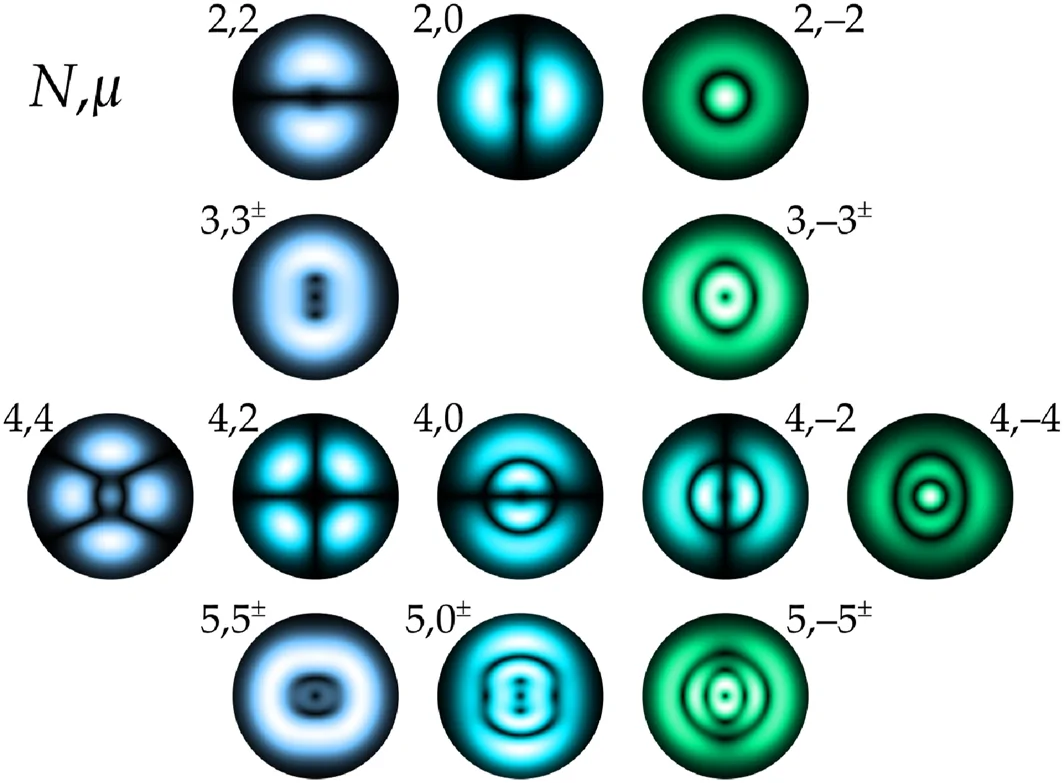
Half‐azimuth EG mode intensities. Intensity patterns for the same modes as in Figure 1, but with azimuth ϕ replaced by ϕ/2. This desymmetrizes the rotationally symmetric EG modes, and this representation brings out the polhode‐like geometry of the nodal patterns.

The paraxial propagation of EG modes is straightforward, and similar to other structured Gaussian modes [16, 17]. As eigenfunctions of $M^{2}$, they spread hyperbolically but with self‐similar intensity patterns, acquiring a Gouy phase of N+1 and, if N is odd, other phase terms consistent with conservation of orbital angular momentum.

## 4 Discussion and Outlook

We have introduced and described the Euler‐top Gaussian (EG) modes, a new family of structured Gaussian beams obtained from the simple quadratic operator $\hat{K}=4\left(J_{3}^{2}-J_{1}^{2}\right)$. Unlike the well‐known HG, LG, and IG mode families, which arise from separation of variables in Cartesian, cylindrical, and elliptic coordinates, the EG modes are motivated algebraically from the operator structure of the 2D harmonic oscillator. Their amplitude structure reflects the quantisation of polhode curves of the Euler top: LG‐like (HG‐like) modes correspond to the stable 3‐axis (1‐axis) rotations and the κ=0 modes to the four‐branch separatrix associated with the intermediate‐axis instability.

In the semiclassical ray‐orbital sphere picture of structured Gaussian modes, each mode is a smooth superposition of SU(2) coherent states lying along a Poincaré path, weighted by the geometric phase. Gaussian mode families correspond to closed contours of functions $F\left(M_{+},M_{\times },L\right)$ on the Gaussian ray‐orbital sphere. Linear functions of the corresponding pseudo‐AM operators yield the LG, HG and GG modes (with quantised solid angle); the Ince operator $\hat{I}$ provides a more complicated, offset cylindrical quadratic. The EG operator $\hat{K}$ is the simplest balanced quadratic combination of Fradkin–Stokes pseudospin operators, and produces the most symmetric nonlinear family of modes, also realising separatrix states at the topological transition.

Among polynomial functions of the Stokes–Fradkin pseudo‐AM SU(2) generators, quadratic observables are the simplest whose contours remain simple closed curves on the ray‐orbital sphere. Up to SU(2) rotations and linear perturbations, the EG modes therefore complete the quadratic algebraic classification of families of structured Gaussian modes. Other choices of Poincaré paths are of course possible, but choices where F is a linear or quadratic combination of Stokes–Fradkin parameters are the only ones for which the structure of the quantised mode family is semiclassically related to the contours of F.

Other Gaussian‐related beams, such as Bessel–Gauss and Mathieu–Gauss beams, are not eigenfunctions of a natural operator related to SU(2) observables, are not eigenfunctions of the beam quality and have no well‐defined mode order, and therefore do not lie within this SU(2) hierarchy of Gaussian beams of fixed beam quality.

Although we have focused on EG modes with K balanced between the 3‐ and 1‐axes, with a transition between squared LG and squared HG modes, the calculations can easily be adapted to eigenfunctions of the operators $J_{1}^{2}-J_{2}^{2}$ and $J_{2}^{2}-J_{3}^{2}$ and beyond, by substituting the LG mode expansion with a suitable family of GG modes. These share similar features to the EG modes described here, such as the topological transition and fourfold symmetric modes which are deformations of squared GG modes, reflecting the polhodes about the rotated inertia ellipsoid in 1,2,3‐space.

Ince–Gaussian modes demonstrate the nonlinear pendulum dynamics of the Ince oscillator, corresponding to the Bose–Hubbard dimer model of condensed matter physics [26]. A similar analogy relates EG modes, through the balanced quadratic operator $\hat{K}$, to a quadrupolar *Lipkin–Meshkov–Glick model*, with zero external field [37, 38]. Ref. [37] considers explicitly the case with a nonzero field term. In the language of this paper, adding $J_{2}$ times a real constant to $\hat{K}$, distorting the dihedral symmetry of the polhodes and destroying the fourfold symmetry of the pure EG modes (whilst retaining a 2‐fold symmetry). Its classical trajectories are precisely the polhodes of the Euler top, with stable and unstable axes and a separatrix [39]. Thus, IG and EG modes provide optical realisations of two of the most important integrable few‐mode many‐body models.

Separation of variables of the 3D Helmholtz equation in sphero‐conical coordinates yields Lamé functions connected with the classical Euler top [32, 40]. However, the 2D harmonic oscillator underlying paraxial Gaussian modes is only separable in Cartesian, polar and elliptic coordinate systems [8], corresponding exactly to the HG, LG and IG families. Therefore, no separable Lamé‐type Gaussian family exists, and the EG modes arise from operator algebra rather than coordinate separability.

EG modes should be straightforward to generate in the laboratory, using standard holographic methods such as computer‐controlled spatial light modulators as superpositions of LG modes. Unlike GG and IG modes [26, 41, 42], EG modes are not obviously generated as cavity modes or by the action of any natural optical element. Their inherent fourfold symmetry makes them an appropriate working eigenbasis whenever π/2‐rotation invariance is required; unlike the ${HG}_{nn}$ subfamily, EG modes form a complete basis of π/2‐symmetric beams. This might include square arrays of optical vortices (especially for odd N and $\kappa_{N,\mu <0}$), stable modes of resonators with quadrupolar mirrors, and applications that require asymmetric orbital angular momentum distributions. Furthermore, their balanced quadratic structure also makes them promising for simulating LMG‐like spin models and topological transitions in tabletop optical experiments.

The EG modes highlight the possibility of new families of optical modes, motivated as eigenfunctions of operators arising in wave‐optical mechanics, beyond the separation of variables approach. Classical angular momentum systems provide a natural setting for the pseudo‐AM operators of structured Gaussian modes: various rotors and tops, mixed products such as $J_{1}J_{3}$ and higher‐order polynomial observables may yield further interesting mode families. More generally, our treatment of the EG modes is an example of a broader programme of structured light in terms of geometric quantisation.

## Author Contributions

**Mark R. Dennis:** investigation, funding acquisition, writing – original draft, validation, visualization, writing – review and editing, formal analysis, project administration, supervision, conceptualization, methodology. **Kerr Maxwell:** investigation, interpretation, writing – review and editing.

## Data Availability

Data sharing not applicable to this article as no datasets were generated or analysed during the current study.

## References

[1] A. Forbes , M. De Oliveira , and M. R. Dennis , “Structured Light,” Nature Photonics 15, no. 4 (2021): 253–262, 10.1038/s41566-021-00780-4.

[2] H. Rubinsztein‐Dunlop , A. Forbes , M. V. Berry , et al., “Roadmap on Structured Light,” Journal of Optics 19, no. 1 (2017): 013001, 10.1088/2040-8978/19/1/013001.

[3] L. Allen , M. W. Beijersbergen , R. J. C. Spreeuw , and J. P. Woerdman , “Orbital Angular Momentum of Light and the Transformation of Laguerre–Gaussian Laser Modes,” Physical Review A 45, no. 11 (1992): 8185–8189, 10.1103/PhysRevA.45.8185. 9906912

[4] S. Danakas and P. K. Aravind , “Analogies Between Two Optical Systems (Photon Beam Splitters and Laser Beams) and Two Quantum Systems (The Two‐Dimensional Oscillator and the Two‐Dimensional Hydrogen Atom),” Physical Review A 45, no. 3 (1992): 1973–1977, 10.1103/physreva.45.1973. 9907186

[5] M. Feldmann , “Modes in General Gaussian Optical Systems Using Quantum‐Mechanics Formalism,” Journal of the Optical Society of America A: Optics and Image Science 61, no. 4 (1971): 446–449, 10.1364/JOSA.61.000446.

[6] G. Nienhuis and L. Allen , “Paraxial Wave Optics and Harmonic Oscillators,” Physical Review A 48, no. 1 (1993): 656–665, 10.1103/PhysRevA.48.656. 9909640

[7] A. M. Yao and M. J. Padgett , “Orbital Angular Momentum: Origins, Behavior and Applications,” Advances in Optics and Photonics 3, no. 2 (2011): 161–204, 10.1364/AOP.3.000161.

[8] C. P. Boyer , E. G. Kalnins , and W. Miller Jr. , “Lie Theory and Separation of Variables: VI. The Equation *iU* _t_ + ∆2*U* = 0,” Journal of Mathematical Physics 16, no. 3 (1975): 499–511, 10.1063/1.522573.

[9] C. P. Boyer , E. G. Kalnins , and W. Miller Jr. , “Lie Theory and Separation of Variables. VII. The Harmonic Oscillator in Elliptic Coordinates and Ince Polynomials,” Journal of Mathematical Physics 16, no. 3 (1975): 512–517, 10.1063/1.522574.

[10] A. E. Siegman , Lasers (University Science Publishers, 1986).

[11] M. A. Bandres and J. C. Gutiérrez‐Vega , “Ince–Gaussian Beams,” Optics Letters 29, no. 2 (2004): 144–146, 10.1364/OL.29.000144. 14743992

[12] R. Gutiérrez‐Cuevas , M. R. Dennis , and M. A. Alonso , “Ray and Caustic Structure of Ince–Gauss Beams,” New Journal of Physics 26, no. 1 (2024): 013011, 10.1088/1367-2630/ad17dc.

[13] E. T. Whittaker , A Treatise on the Analytical Dynamics of Particles and Rigid Bodies (Cambridge University Press, 1917).

[14] H. Goldstein , Classical Mechanics, 2nd ed. (Addison‐Wesley Publishing Company, 1980).

[15] M. R. Dennis and M. A. Alonso , “Swings and Roundabouts: Poincaré Spheres for Polarization and Gaussian Beams,” Philosophical Transactions of the Royal Society A 375, no. 2087 (2017): 20150441, 10.1098/rsta.2015.0441. PMC5247485 28069772

[16] M. A. Alonso and M. R. Dennis , “A Ray‐Optical Poincaré Sphere for Structured Gaussian Beams,” Optica 4 (2017): 476–486, 10.1364/OPTICA.4.000476.

[17] M. R. Dennis and M. A. Alonso , “Gaussian Mode Families From Systems of Rays,” Journal of Physics: Photonics 1, no. 2 (2019): 025003, 10.1088/2515-7647/ab011d.

[18] R. Gutiérrez‐Cuevas , M. R. Dennis , and M. A. Alonso , “Generalized Gaussian Beams in Terms of Jones Vectors,” Journal of Optics 21, no. 8 (2019): 084001, 10.1088/2040-8986/ab2c52.

[19] J. Schwinger , “On Angular Momentum,” in Quantum Theory of Angular Momentum, L. C. Biedenharn and H. van Dam , eds. (Academic Press, 1965), 229–279.

[20] M. J. Padgett and J. Courtial , “Poincaré‐Sphere Equivalent for Light Beams Containing Orbital Angular Momentum,” Optics Letters 24, no. 7 (1999): 430–432, 10.1364/OL.24.000430. 18071529

[21] G. S. Agarwal , “SU(2) Structure of the Poincaré Sphere for Light Beams With Orbital Angular Momentum,” Journal of the Optical Society of America A 16, no. 12 (1999): 2914–2916, 10.1364/JOSAA.16.002914.

[22] E. G. Abramochkin and V. G. Volostnikov , “Generalized Gaussian Beams,” Journal of Optics A: Pure and Applied Optics 6, no. 5 (2004): S157–S161, 10.1088/1464-4258/6/5/001.

[23] S. J. M. Habraken and G. Nienhuis , “Universal Description of Geometric Phases in Higher‐Order Optical Modes Bearing Orbital Angular Momentum,” Optics Letters 35, no. 20 (2010): 3535–3537, 10.1364/OL.35.003535. 20967125

[24] Y. Shen , “Rays, Waves, SU(2) Symmetry and Geometry: Toolkits for Structured Light,” Journal of Optics 23, no. 12 (2021): 124004, 10.1088/2040-8986/ac3676.

[25] K. B. Wolf , Geometric Optics on Phase Space (Springer, 2004).

[26] R. Gutiérrez‐Cuevas , D. H. J. O’Dell , M. R. Dennis , and M. A. Alonso , “Exactly Solvable Model Behind Bose‐Hubbard Dimers, Ince‐Gauss Beams, and Aberrated Optical Cavities,” Physical Review A 107, no. 3 (2023): L031502, 10.1103/PhysRevA.107.L031502.

[27] L. Poinsot , “Théorie Nouvelle de la Rotation des Corps,” Journal de Mathematiques Pures et Appliquees 16 (1851): 9–129, www.numdam.org/item?id=JMPA_1851_1_16__9_0.

[28] M. V. Berry , “The Adiabatic Phase and Pancharatnam’s Phase for Polarized Light,” Journal of Modern Optics 34, no. 11 (1987): 14–17, 10.1080/09500348714551321.

[29] N. M. J. Woodhouse , Geometric Quantization, 2nd ed. (Oxford University Press, 1998).

[30] K. Maxwell , “Structured Gaussians From Geometric Quantisation,” preprint (2025), https://arxiv.org/abs/2503.20775.

[31] M. J. Gotay , H. Grundling , and C. A. Hurst , “A Groenwald‐Van Hove Theorem for S^2^ ,” Transactions of the American Mathematical Society 348, no. 4 (1996): 1579–1597, 10.1090/S0002-9947-96-01559-0.

[32] J. Patera and P. Winternitz , “A New Basis for the Representations of the Rotation Group. Lamé and Heun Polynomials,” Journal of Mathematical Physics 14, no. 8 (1973): 1130–1139, 10.1063/1.1666449.

[33] M. Fernandez Guasti , “Analytic Geometry of Some Rectilinear Figures,” International Journal of Education, Technology and Science 23 (1992): 895–901, 10.1080/0020739920230607.

[34] H. Fong , “Squircular Calculations,” preprint (2016), https://arxiv.org/abs/1604.02174v1.

[35] L. C. Biedenharn and J. D. Louck , Angular Momentum in Quantum Physics (Cambridge University Press, 2010).

[36] J. J. Sakurai , Modern Quantum Mechanics, Revized Edition (Addison‐Wesley, 1993).

[37] H. J. Lipkin , N. Meshkov , and A. J. Glick , “Validity of Many‐Body Approximation Methods for a Solvable Model,” Nuclear Physics 62, no. 2 (1965): 188–198, 10.1016/0029-5582(65)90862-X.

[38] N. Debergh and F. Stancu , “On the Exact Solutions of the Lipkin–Meshkov–Glick Model,” Journal of Physics A: Mathematical and General 34, no. 15 (2001): 3265–3276, 10.1088/0305-4470/34/15/305.

[39] T. Opatrný , L. Richterek , and M. Opatrný , “Analogies of the Classical Euler Top With a Rotor to Spin Squeezing and Quantum Phase Transitions in a Generalized Lipkin–Meshkov–Glick Model,” Scientific Reports 8, no. 1 (2018): 1984, 10.1038/s41598-018-20486-y. PMC5792583 29386576

[40] I. Lukach and Y. A. Smorodinskii , “Separation of Variables in a Spheroconical Coordinate System and the Schrödinger Equation for a Case of Noncentral Forces,” Theoretical and Mathematical Physics 14 (1973): 125–131, 10.1007/BF01036350.

[41] A. T. O’Neil and J. Courtial , “Mode Transformations in Terms of the Constituent Hermite–Gaussian or Laguerre–Gaussian Modes and the Variable‐Phase Mode Converter,” Optics Communications 181, no. 1–3 (2000): 35–45, 10.1016/S0030-4018(00)00736-7.

[42] U. T. Schwarz , M. A. Bandres , and J. C. Gutiérrez‐Vega , “Observation of Ince–Gaussian Modes in Stable Resonators,” Optics Letters 29, no. 16 (2004): 1870–1872, 10.1364/OL.29.001870. 15357343

